# Theoretical Calculation and Experimental Studies of Boron Phosphide Polycrystalline Synthesized at High Pressure and High Temperature

**DOI:** 10.3390/nano15060446

**Published:** 2025-03-15

**Authors:** Peng Yang, Ziwei Li, Haidong Yu, Shan Gao, Xiaopeng Jia, Hongan Ma, Xilian Jin

**Affiliations:** 1 State Key Laboratory of High Pressure and Superhard Materials, College of Physics, Jilin University, Changchun 130012, China; yp15116285837@163.com (P.Y.); lizw23@mails.jlu.edu.cn (Z.L.); yhd19971012@163.com (H.Y.); gaoshanvip827@126.com (S.G.); jiaxp@jlu.edu.cn (X.J.); 2 College of Physics and Electronic Information Engineering, Tongren University, Tongren 554300, China

**Keywords:** boron phosphide, crystal structure, electrical properties, theoretical calculations, HPHT synthesis

## Abstract

In this study, a combination of theoretical calculations and experiments were carried out to analyze boron phosphide materials. Amorphous boron powder and amorphous red phosphorus were used as raw materials to directly synthesize the target samples in one step under high-pressure and high-temperature (HPHT) conditions. Theoretical calculations were then carried out based on the XRD spectra of boron phosphide at 4 GPa and 1200 °C. The experimental results show that the target samples can be successfully prepared at HPHT. The electrical properties of the samples were characterized, and it was found that their conductivity increased with the increase in temperature, and they have a semiconducting nature, which is consistent with the theoretical calculations. Its Seebeck coefficient is positive at different temperatures, indicating that the synthesized boron phosphide is a P-type semiconductor. The combination of theoretical calculations and experiments shows that high pressure can reduce the lattice constant of boron phosphide, thus reducing its forbidden bandwidth, which improves its electrical properties. EDS shows a homogeneous distribution of the elements in the samples. Successful synthesis of BP crystals will probably stimulate more research into its semiconductor properties. It may also provide some assistance in the application of BP in aero-engine high-temperature monitoring systems as well as thermally controlled coatings for deep-space probes.

## 1. Introduction

The study of binary compounds at high pressures has attracted a great deal of attention from scientists and researchers in the last few decades, thanks to their potentially excellent properties, such as high-temperature superconductivity, very high hardness and resistance to metallization [1,2,3,4,5,6,7,8,9]. Because BP exhibits typical semiconductor temperature-sensitive properties, the jump in such properties stems from the high-voltage-induced coupling of strong sp^3^ hybridized orbitals and the suppression of lattice distortions, which facilitates the application of BP in the subsequent industrial scenarios: high-temperature and extreme-environmental electronics, where the broad bandwidth and thermal stability of BP make it suitable for high-temperature environmental applications [10,11,12,13]. HPHT-synthesized polycrystalline materials of BP show potential for high-temperature sensors. BP films have a transmittance of more than 90% in the infrared band and have anisotropic light absorption properties, making them suitable for infrared detection and thermal management. As thermal control coatings for deep-space probes, BP films can be adjusted in thickness to achieve dynamic infrared radiation modulation, thus improving the reliability of devices in extreme temperature environments [13]. However, the main bottlenecks in the industrialization of BP are the high cost of large-scale preparation and the lack of environmental stability (susceptible to oxidation). Future research could focus on encapsulation technologies—development of atomic layer deposition (ALD) protective layers to extend the lifetime of BP devices—and doping modifications—modulation of forbidden bandwidth and carrier concentration by elemental doping (e.g., sulfur and selenium) [13,14]. In the dynamic landscape of materials science, boron phosphide has emerged as a prominent material of interest, primarily due to its exceptional hardness and superior thermal conductivity, which render it highly promising for advanced technological applications [15,16]. In advanced HPHT synthesis of boron phosphide, high-quality BP can be obtained through effective suppression of decomposition reactions and precise modulation of lattice dynamics. However, comprehensive investigations into the direct transformation pathways of amorphous boron and phosphorus precursors and the impact of residual stress on electrical transport characteristics remain underexplored, which are critical for optimizing device performance [10,17]. There are several methods for synthesizing boron phosphide materials; CVD synthesis is a method that requires a long cycle time for synthesis and uses reactants that are hazardous gases and require special treatment of the off-gas before it can be released into the air. The synthesis of boron phosphide using the solute growth method also requires a long cycle time, and the decomposition product of boron phosphide (B_12_P_2_) is found in the product. Therefore, the preparation of BP with high efficiency and quality is still a great challenge for conventional synthesis methods. However, boron phosphide is a very stable phase under high pressure, and the decomposition phase of boron phosphide (B_12_P_2_) does not exist; in addition, the cycle time for the preparation of BP under high temperature and high pressure is very short, which can be obtained by heating for only 1 h. Meanwhile, the preparation of BP using amorphous boron powder and amorphous red phosphorus as raw materials under high temperature and high pressure has rarely been mentioned in previous reports. The above-mentioned conventional synthesis methods are limited by the sub-stability properties of BP at atmospheric pressure and generally face challenges such as many crystal defects and difficulty in suppressing the decomposition product B_12_P_2_, which leads to a significant degradation of the electrical properties of the material [18,19]. Therefore, the aim of the present work is to explore a new method to synthesize boron phosphide under high pressure with high efficiency, as well as to explore the preparation process of boron phosphide and the electrical properties of boron phosphide as a semiconductor material. In this study, we broke through the limitation of traditional raw materials and synthesized high-purity BP polycrystalline materials for the first time by one-step HPHT solid-state reaction (4 GPa/1200 °C) using amorphous boron powder and amorphous red phosphorus as raw materials. Combined with XRD spectra and theoretical calculations, it is found that the high pressure has a certain mechanism to regulate the structural and electrical properties of BP: the lattice constant becomes narrower, and the forbidden bandwidth decreases.

Therefore, the focus of this study is the one-step synthesis of boron phosphide materials using amorphous boron powder and amorphous phosphorus powder under HPHT conditions, as well as the theoretical calculation and characterization of their electrical properties. The main objective of this study is to systematically investigate the preparation of boron phosphide polycrystalline materials using amorphous boron and phosphorus sources under HPHT conditions, followed by theoretical calculations from the XRD spectra of boron phosphide at 4 GPa and 1200 °C. The calculated results are in agreement with the actual experimental results, revealing that the electrical properties of boron phosphide materials can be improved using HPHT, which is attributed to the fact that high pressure can be used to reduce the lattice constants and forbidden bandwidths of boron phosphide.

In this context, boron phosphide ceramic materials were successfully synthesized by an HPHT solid-phase method in this study. The target samples were successfully synthesized under different pressure and temperature conditions. The XRD patterns of boron phosphide at 4 GPa and 1200 °C were then selected for theoretical calculations. The high-temperature and high-pressure synthesis method has many advantages, such as maintaining the specific properties of the samples from high pressure to atmospheric pressure, improving the electrical properties of the materials and nanosizing the sample materials. Through experiments and theoretical calculations, our results show that the target samples can be successfully prepared at HPHT, and their electrical properties were characterized, and their conductivity increases with increasing temperature, which is consistent with the nature of semiconductors. Although we found that the lattice constant and forbidden bandwidth of BP can be reduced by using amorphous boron powder and amorphous red phosphorus under high pressure through theoretical calculations, we found that the conductivity enhancement of BP is not obvious in our experiments.

## 2. Calculation Method

The calculations were carried out using the first-principles density functional theory (DFT). The Vienna Ab Initio Simulation Package (VASP) was employed for the calculations, including structure optimization, electronic band structure and electronic localization function [20]. The Perdew–Burke–Ernzerhof (PBE) of the generalized gradient approximation (GGA) was chosen as the exchange–correlation functional [21]. The projected augmented-wave (PAW) pseudopotential was used for both B and P atoms, and 2s^2^2p^1^ and 3s^2^3p^3^ were considered as valence electrons for B and P, respectively [22]. A plane-wave basis set with an energy cutoff of 450 eV and k-point grid spacing of 2π × 0.03 Å^−1^ was adopted in this work. The convergence criteria for the atomic force and total energy were set at 10^−5^ eV/Å and 10^−6^ eV per atom, respectively. To accurately calculate the electronic structures, the Heyd−Scuseria−Ernzerhof (HSE06) hybrid functional was employed to treat the electron exchange−correlation functional [23]. Based on density functional perturbation theory (DFPT), phonon calculations were conducted through the Phonopy code [24]. And the 3 × 3 × 3 supercell containing 54 atoms was chosen for phonon calculations.

## 3. Experimental Section

**Figure 1:**
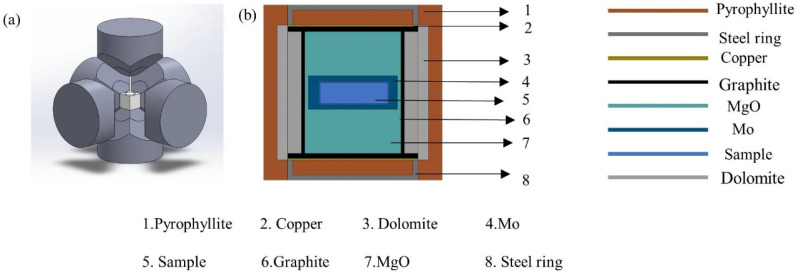
(**a**) Structure of the Chinese Hexagonal Top High Pressure Device. (**b**) Assembly diagram of the sample.

Purchased amorphous boron powder (B) and phosphorus powder (P) were used as raw materials (not further purified); the weighed powders were weighed according to the stoichiometric ratio, and the raw materials were mixed thoroughly for 1 h, and then pressed into cylinders with a diameter of 10.5 mm and a thickness of 4.5 mm using a cold press mold. Before the start of the experiment, the cylinder was put into the composite block, and molybdenum foil was used to wrap the cylinder in order to prevent the assembled parts from contaminating the raw materials. Before the experiment, the prepared cylindrical raw material was put into the composite block, and the assembled composite block was put into the Chinese Hexagonal Top High Pressure Device ((CHPA) SPD − 6 × 1200) shown in Figure 1a for sintering. The picture of the assembly is shown in Figure 1b. The synthesis conditions of the samples were then determined. The synthetic heating time was 60 min at a pressure condition of 4.0 GPa and a temperature interval of 1050 °C to 1350°. Using the above steps, the target samples were successfully synthesized by rapidly quenching the samples under high pressure to ambient temperature before depressurization. The chamber was uniformly heated by graphite tubing, while the assembly was energized and simultaneously pressurized by a hydraulic multi-anvil technique. A reference material was also used to calibrate the pressure, as pressure induces a phase transition in the reference material at a given pressure. A Pt-RH/Pt-Rh type 6 thermocouple contact Cali was used at the sample location for working temperature. The internal temperature of the chamber was rapidly quenched to room temperature before unloading the pressure.

## 4. Characterization

The diameter of the sintered sample is about 10 mm, and the thickness is about 4.5 mm. First, the surface of the sample is polished with sandpaper. In order to examine the phase structure of the samples, XRD spectra are usually collected using an X-ray diffractometer, which is a Rigaku SmartLab SE (Tokyo, Japan) with a range of 30–90° and a scanning step of 0.01°. The obtained XRD spectra are analyzed by professional software, such as Jade software (jade6.5). These software programs can be compared with standard crystal structure databases to determine the phase structure of the samples. The morphological characteristics of the fracture surfaces of the samples were also measured using a field emission scanning electron microscope (SEM, FEI Magellan, Hillsboro, OR, USA). The variable temperature electrical properties of the thermoelectric samples were tested using a JouleYacht Namicro-IIIL thermoelectric test system (Jiayitong, Sshenzhen, China).

## 5. Results and Discussion

**Figure 2:**
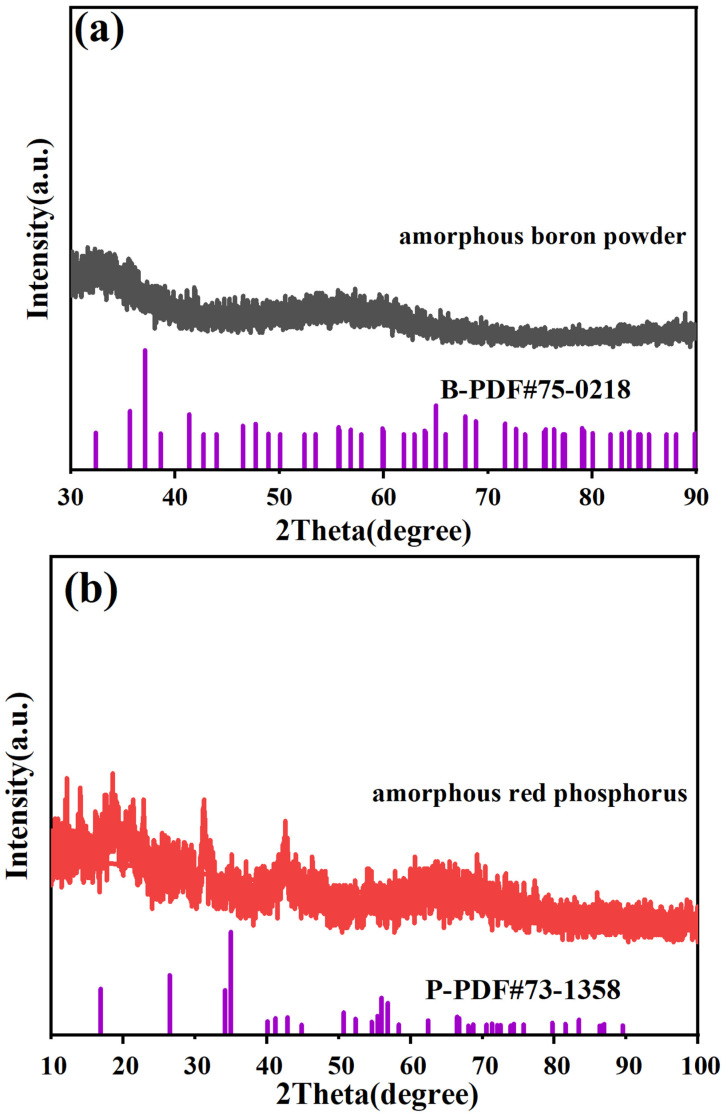
XRD diffraction patterns of raw materials used for synthesizing BP. (**a**) XRD diffraction pattern of amorphous boron powder. (**b**) XRD diffraction pattern of amorphous red phosphorus.

The samples we used were amorphous boron source and phosphorus source, i.e., amorphous boron powder and amorphous red phosphorus. The XRD spectra of amorphous boron powder and amorphous red phosphorus are shown in Figure 2a, and the XRD spectra of amorphous red phosphorus are shown in Figure 2b. From the XRD spectra, we found that the raw materials used do not have any obvious diffraction peaks, and they are diffusely elevated, so that the boron source and phosphorus source used are amorphous. Therefore, the boron and phosphorus sources used are amorphous. However, we succeeded in synthesizing crystalline boron phosphide materials in one step using an HPHT method, and prepared boron phosphide BP materials with a crystalline structure and distinct diffraction peaks. At the same time, we used amorphous boron and phosphorus sources, which require more relaxed conditions than using crystalline raw materials to synthesize boron phosphide. Therefore, the use of amorphous boron and phosphorus sources allows the one-step synthesis of boron phosphide crystals at high temperatures and pressures in a very simple way.

**Figure 3:**
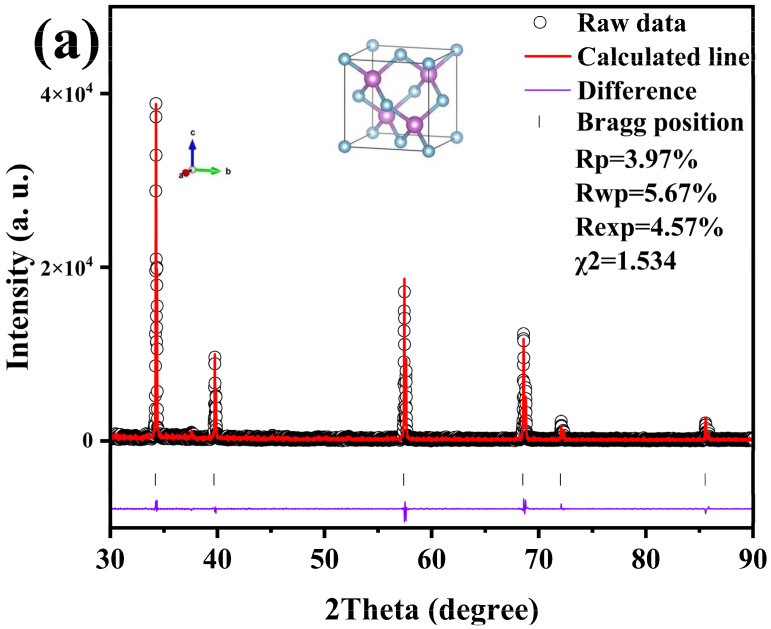

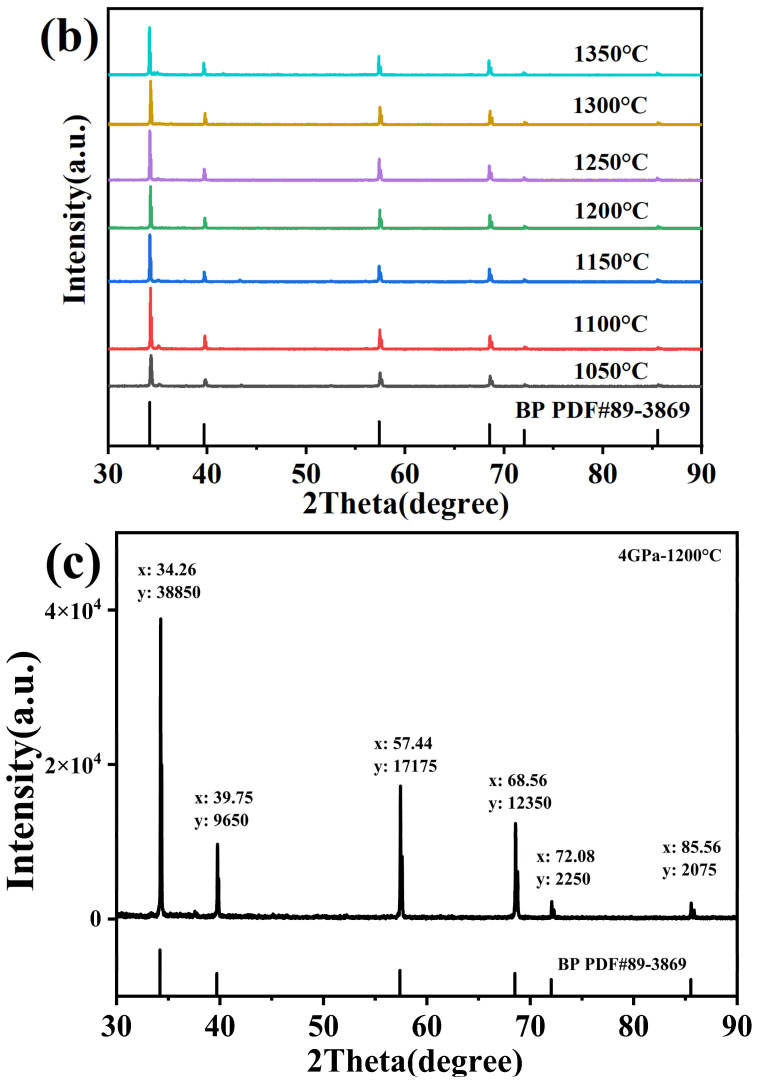
(**a**) XRD refinement of BP synthesized at a pressure of 4 GPa and at 1200 °C and crystal structure of BP. (**b**) X-ray diffraction patterns of BP synthesized at a pressure of 4 GPa and different synthesis temperatures. (**c**) Raw XRD of BP synthesized at a pressure of 4 GPa and at 1200 °C and crystal structure of BP.

The results of Rietveld refining of boron phosphide (BP) prepared by the HPHT method at 4 GPa and 1200 °C are shown in Figure 3a with a good fit. Figure 3a shows the X-ray diffraction (XRD) refinement results of boron phosphide (BP) at 4 GPa and 1200 °C. The horizontal axis 2 Theta (degrees) indicates the diffraction angle. Vertical axis: Intensity (a.u.) indicates the intensity of the diffraction peaks. Black circles indicate raw data points. The red line indicates the calculated fitting curve. The purple line indicates the difference between the raw data and the fitted curve. The results of the fit show an Rp of 3.97%, indicating the fitted residual coefficient, an rwp of 5.67%, indicating the weighted residual coefficient, and an rexp of 4.57%, indicating the experimental residual coefficient. A χ^2^ of 1.534 indicates a good fit. The figure shows several distinct diffraction peaks located at approximately 30°, 40°, 60° and 70°, respectively. The positions of these peaks correspond to the crystal structure of boron phosphide, indicating that the sample is well crystallized. The crystal structure of boron phosphide is modeled in the inset of Figure 3a, where the p-atoms are light blue and the b-atoms are purple. A cubic crystal cell is shown, indicating that boron phosphide has a cubic crystal system. The surface synthesized boron phosphide has a sphalerite structure, and it can be seen that the final product of boron phosphide (BP) is a pure phase with a sphalerite structure and a space group of *F*-43m (No. 216), which is structurally homogeneous with most zinc blende materials [25]. The XRD spectra of boron phosphide at 4 GPa and 1200 °C show good crystallinity with well-defined and accurately located diffraction peaks. The fitting results show that the calculated fitting curves are in good agreement with the experimental data, and the residual coefficients are small, indicating a good fitting effect. The crystal structure of boron phosphide is a cubic crystal system, and the positions of P and B atoms in the crystal cell are clear. Figure 3b shows the X-ray diffraction patterns at different temperatures under the pressure of 4 GPa. The synthesis times were all 60 min. The XRD patterns at 4 GPa are shown along with the standard boron phosphide PDF pattern. The standard plot shows the diffraction peak positions and intensities of boron phosphide under standard conditions. As shown, there are six major diffraction peaks. As can be seen from the figure, the target samples of boron phosphide (BP) have been successfully synthesized at different temperature conditions. Therefore, amorphous boron powder and amorphous red phosphorus can be directly synthesized into boron phosphide (BP) in one step under HPHT conditions with a very simple preparation process, which indicates that the HPHT method for the preparation of boron phosphide (BP) has significant advantages. Figure 2b shows the successful synthesis of the target samples at different synthesis temperatures under 4 GPa. It can be seen that when the sintering temperature is low, the samples synthesized at 1050 °C and 1100 °C have obvious heterogeneous peaks near 35°, and as the temperature increases, the heterogeneous phases are weaker when the synthesis temperature is close to 1200 °C, and they continue to increase to 1300 °C, where there is almost no heterogeneous phase. The samples synthesized at 1050 °C and 1100 °C also showed obvious heterogeneous peaks near 35°. This indicates that there is a clear positive correlation between the purity and temperature when synthesizing boron phosphide materials at HPHT. Figure 3c shows the raw XRD data at 4 GPa and 1200 °C, and the theoretical calculations were based on these XRD data. All the XRD patterns that can be seen have double peaks, i.e., each peak is overlapped by another peak of half its intensity, which is a normal phenomenon due to the use of XRD test equipment for double-ray testing. This is related to the copper target of the XRD test equipment, which has two wavelengths, Cu Kα1 at 1.5406 Å and Cu Kα2 at 1.5444 Å. The two wavelengths are very close to each other, with an intensity ratio of about two to one. When the crystal structure is more perfect and the diffraction peaks are narrower, the peaks of the two wavelengths may be separated, resulting in the appearance of the double peaks on the XRD patterns. Meanwhile, we successfully synthesized the target samples of boron phosphide materials at 2, 3, 4 and 5 GPa.

**Figure 4:**
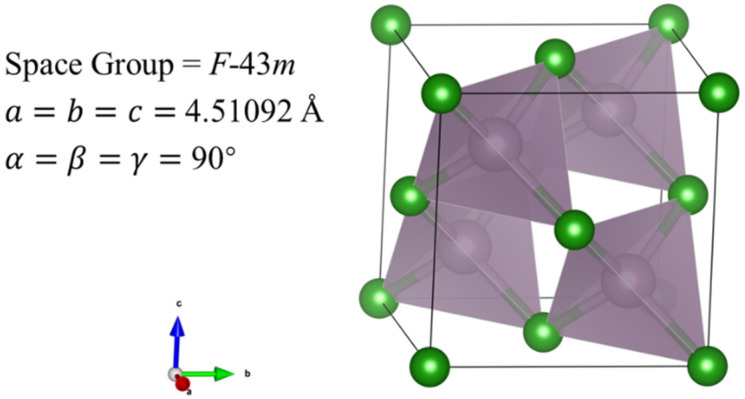
Crystal structure of BP compounds with space group *F*-43m calculated based on XRD pattern at 4 GPa. Purple and green spheres depict P and B atoms, respectively. As shown in Figure 4, P atoms are coordinated by four B atoms in a tetrahedral form with a distance between B and P atoms of 1.95329 Å.

**Figure 5:**
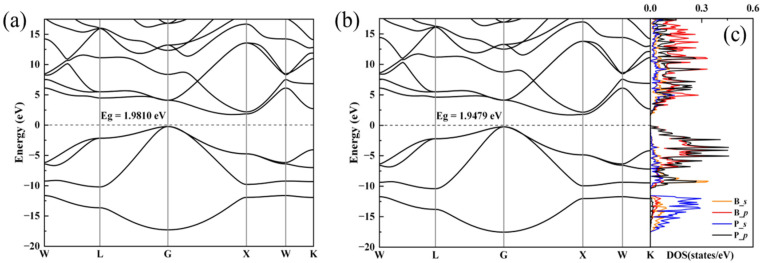
Electronic properties for *F*-43m BP calculated based on XRD pattern at 1 bar and 4 GPa. (**a**,**b**) Band structures of *F*-43m BP at 1 bar and 4 GPa, respectively. (**c**) The partial density of states for *s* and *p* orbitals of B and P atoms at 4 GPa.

To further elucidate the structure and properties of the BP compounds, we theoretically calculated the electronic and phonon structures of *F*-43m BP. Under the pressure condition of 1 bar and 4 GPa, we optimized the *F*-43m structure provided by a PDF standard card with highly precise geometry optimization; the optimized crystal structure and structural information are displayed in Figure 4 and Table 1, respectively. Band structures for the optimized *F*-43m structure at 1 bar and 4 GPa are shown in Figure 5a,b, respectively. Our calculations reveal that the conduction band minimum and valence band maximum are located at disparate positions within the k-space, with band gap values of 1.9810 eV at 1 bar and of 1.9479 eV at 4 GPa, suggesting that the BP compounds exhibit the characteristic of an indirect band gap semiconductor. The values of the lattice constant and band gap at 1 bar are very close to those reported previously; however, the values of the lattice constant and band gap at 4 GPa differ significantly from those of boron phosphide synthesized at similar pressure of 5 GPa, as displayed in Table 2. The partial density of states (PDOS) for the *F*-43m structure at 4 GPa are shown in Figure 5c. It is found that the electrons populating the top of the valence band predominantly originate from the B_*p* and P_*p* orbitals. From this analysis, the conductivity of the semiconductor is observed to manifest an increasing trend with a rise in temperature, the cause of which is the migration of *p*-state electrons from the two atoms at the top of the valence band to the bottom of the conduction band, thereby facilitating the transport within the semiconductor.

Based on our calculations above at 1 bar and 4 GPa, as atmospheric pressure is increased to the high pressure of 4 GPa, it can be seen that the lattice constant reduces from 4.54659 Å to 4.51092 Å and the band gap value reduces from 1.9810 eV to 1.9479 eV, which allows us to draw the conclusion that the reduction in the lattice constant of BP samples with rising pressure results in a lowered band gap. Therefore, we reasonably predict that the band gap of BP samples decreases under higher pressures, leading to increased conductivity.

**Figure 6:**
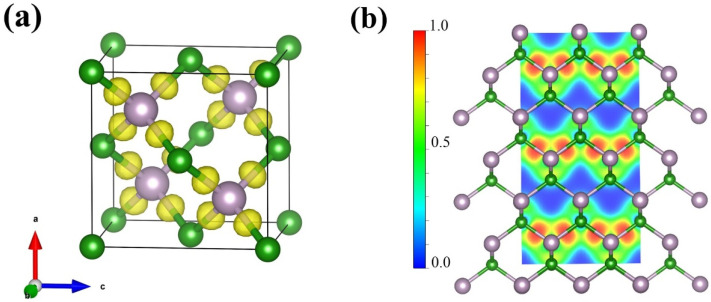
(**a**) Three-dimensional electron localization function (3D ELF) with an isosurface value of 0.75 and (**b**) two-dimensional electron localization function (2D ELF) slice along the (110) plane for *F*-43m BP calculated based on XRD pattern at 4 GPa.

The electron localization function (ELF) is employed to understand the type of bonding and the degree of electronic localization. As demonstrated in Figure 6a,b, the electrons are found to be localized between the boron and phosphorus atoms, leading to the formation of a covalent bond. Notably, the electrons between boron and phosphorus are biased towards the P atoms, thus conferring a polar nature to the covalent bond.

**Figure 7:**
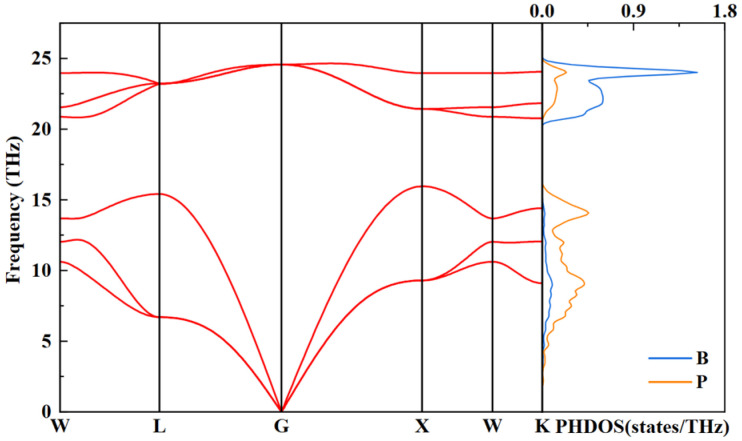
Phonon properties of *F*-43m BP at 4 GPa: (**left panel**) calculated phonon dispersion curves; (**right panel**) projected phonon density of states of B and P atoms.

To determine the dynamical properties of the BP structure, we researched the phonon spectrum and projected phonon density of states for the optimized *F*-43m structure at 4 GPa, as illustrated in Figure 7. Clearly, the phonon dispersion curves do not exist at imaginary frequencies within the first Brillouin zone, indicating the lattice dynamics stability of *F*-43m BP. In addition, the calculation results on the right panel of Figure 7 imply that the vibrational modes of the structure are distributed in the high-frequency and low-frequency regions. In the low-frequency region, the vibrational contribution of the P atoms is larger than that of the B atoms. Conversely, the B atoms contribute more significantly to the vibrations in the high-frequency region. This discrepancy can be ascribed to the fact that the P atoms possess a larger atomic mass than the B atoms.

**Figure 8:**
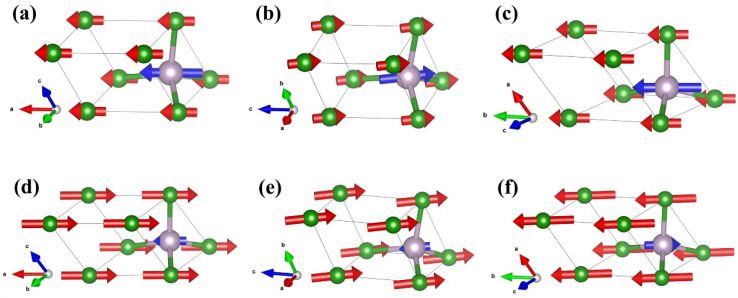
The diagrams of the vibration modes for *F*-43m BP calculated based on XRD pattern at 4 GPa: (**a**–**c**) three acoustic modes; (**d**–**f**) three optical modes. Purple and green spheres depict P and B atoms, respectively. The directions pointed by the red and blue arrows are the vibration directions of B and P atoms, respectively, where the size of the arrows is proportional to the magnitude of the vibration.

As a supplement, the vibrational modes of the *F*-43m structure were subjected to detailed study. The primitive cell of *F*-43m BP comprises one B atom and one P atom, and thus there are six vibrational modes. Γ = Γ_acoustic_ + Γ_optic_ = 3T_2_ + 3T_2_; the acoustic modes and optical modes are both threefold degenerated T_2_ modes. Three acoustic and three optical vibrational modes are shown in Figure 8. In the long-wave approximation, two atoms of *F*-43m BP in the acoustic modes vibrate in the same direction and can be regarded as a collective motion. However, two atoms in the optical modes vibrate in opposite directions.

**Figure 9:**
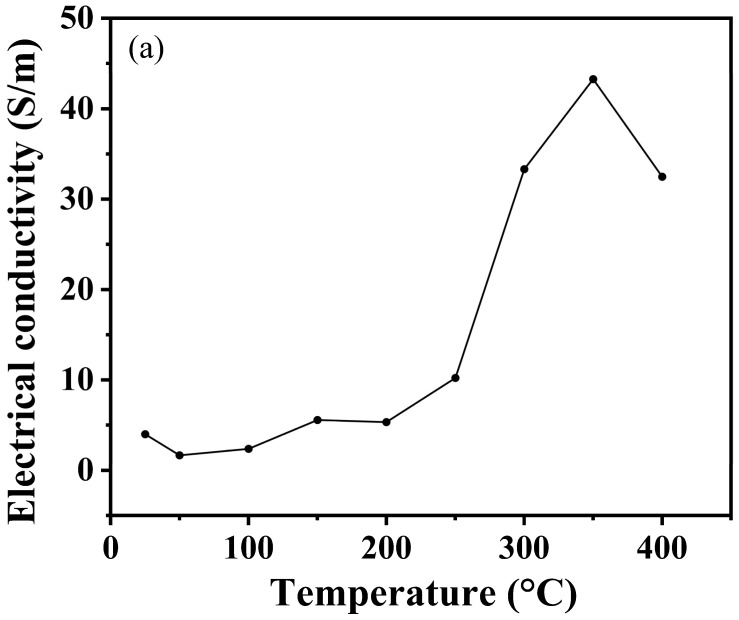

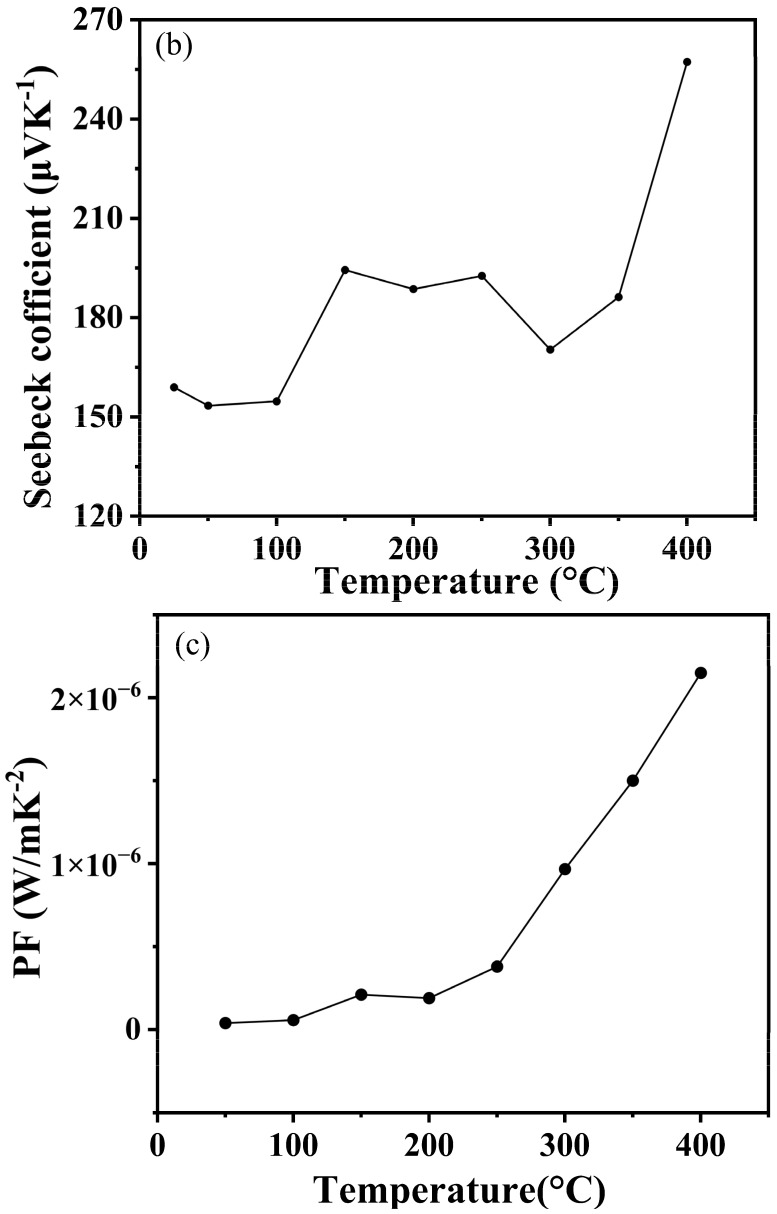
(**a**) Variation in conductivity of HPHT synthesized BP with test temperature. (**b**) Variation in Seebeck coefficients of HPHT synthesized BP with test temperature. (**c**) Variation in power factor of HPHT synthesized BP with test temperature.

As shown in Figure 9a, the conductivity of boron phosphide (BP) samples varies with temperature after synthesis at different temperatures. Overall, it can be seen that the conductivity of boron phosphide shows a clear upward trend with increasing temperature. The reasons for this can probably be analyzed from three aspects: the enhancement of electronic thermal motion, the increase in carrier concentration and the change in crystal structure. Firstly, as the temperature rises, the thermal movement of electrons in boron phosphide will be more intense, thus gaining more energy, and the migration in the crystal lattice will become easier, which makes the conductivity of boron phosphide rise. Secondly, an increase in temperature will generally cause more electrons to jump from the valence band to the conduction band, resulting in an increase in the number of electrons in the conduction band, which will make the concentration of boron phosphide carriers increase, so the conductivity of boron phosphide will also increase. From the crystal structure to be analyzed, the increase in temperature may make the crystal structure of boron phosphide change slightly, and the defects or lattice vibration within the crystal are intensified, which is conducive to the scattering and migration of electrons, thus having a positive impact on the increase in electrical conductivity. Figure 9b shows the temperature dependence of the Seebeck coefficient for boron phosphide synthesized at 4 GPa and 1200 °C. The following is a detailed analysis of the graph: the temperature range in the graph is from 25 °C to 400 °C. The Seebeck coefficient ranges from 160 μV/K to 260 μV/K. The Seebeck coefficient decreases slightly between 25 °C and 100 °C from about 160 μV/K to about 150 μV/K. Between 100 °C and 200 °C, the Seebeck coefficient rises considerably to about 200 °C and 300 °C. Between 100 °C and 200 °C, the Seebeck coefficient fluctuates slightly but generally remains between 180 μV/K and 190 μV/K. Between 300 °C and 400 °C, the Seebeck coefficient rises sharply to about 260 μV/K. At about 100 °C, the Seebeck coefficient begins to rise significantly, indicating that the thermoelectric properties of boron phosphide change significantly in this temperature range. Between 300 °C and 400 °C, the Seebeck coefficient increases sharply, indicating that the thermoelectric properties of boron phosphide are further enhanced in this temperature range. The change in the Seebeck coefficient may be related to the change in electronic structure and energy band structure of boron phosphide with temperature. At high temperatures, the electron mobility and carrier concentration of boron phosphide may change, leading to a significant increase in the Seebeck coefficient. As shown in Figure 9c, which shows the trend of power factor of boron phosphide with temperature, it can be clearly seen that its power factor increases with increasing temperature. This is also in line with the nature of semiconductor materials, where the electrical properties increase with increasing temperature. Overall, the figure shows the trend of the Seebeck coefficient of boron phosphide as a function of temperature at 4 GPa and 1200 °C. The figure shows the temperature dependence of the Seebeck coefficient of boron phosphide for all temperature conditions. The positive values of the Seebeck coefficient at all temperature conditions indicate that the boron phosphide (BP) materials synthesized under these conditions are P-type semiconductors. The sharp increase in the Seebeck coefficient in the temperature range from 350° to 400° coincides with the sudden drop in conductivity at this temperature, which may be related to the fact that the raw material used is an amorphous boron powder, leading to the volatilization of the sample in this temperature range at atmospheric pressure. Meanwhile, we can see that the conductivity is 4 S/m at room temperature, which is an improvement. In addition, the advantage of using amorphous feedstock is the cheap cost and the simplicity of preparation without a precursor compared to using crystalline boron and phosphorus source as feedstock to synthesize boron phosphide.

**Figure 10:**
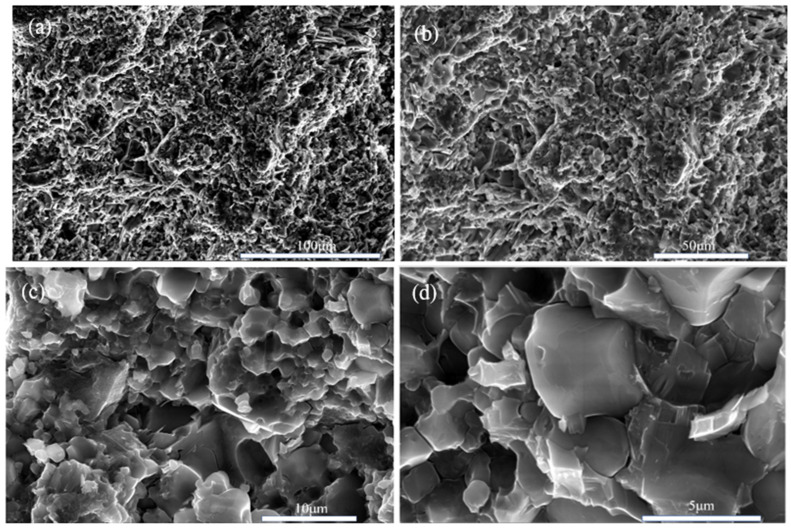

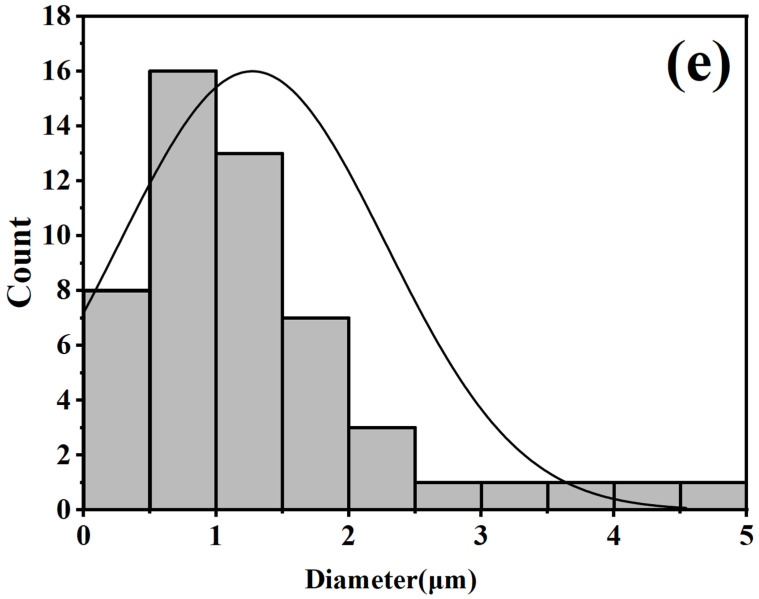
SEM morphology of BP synthesized under HPHT. (**a**) Scale bar is 100 μm; (**b**) scale bar is 50 μm; (**c**) scale bar of 10 μm; (**d**) scale bar of 5 μm; (**e**) BP grain size distribution.

The picture (Figure 10) shows scanning electron microscope (SEM) images of boron phosphide samples synthesized at a pressure of 4 GPa. The images show the fracture surface morphology of the samples at different scales of 100 μm, 50 μm, 10 μm and 5 μm, respectively. From the 100 μm scale image, it can be seen that the morphology of the surface of the sample is rougher, with many irregular concave and convex structures on the surface. In the 50 μm scale image, the details are clearer, and more particles and cracks can be seen. The irregularities on the surface are more obvious, showing the complex structure inside the sample. In the 10 μm scale image, the details are clearer, and smaller particles and cracks can be seen. Surface roughness and irregularities are more pronounced. The 5 μm scale image shows the most detail, and the smallest particles and cracks can be seen. The microstructure of the surface is very clear, showing the complex details inside the sample. From the statistical distribution of grains in Figure 10e, it can be seen that some grains have their diameter sizes between the nanometer scale and the micrometer scale. Figure 10e, it can be seen that there are some grains of a few hundred nanometers in the sample. Overall, the boron phosphide sample has a multi-sized structure under high-pressure 4 GPa conditions. These SEM images demonstrate the fractured surface morphology of the boron phosphide samples at different scales, indicating the presence of complex microstructures and irregular surface morphology inside the samples. This information is important for understanding the physical and chemical properties of the material and its applications.

**Figure 11:**
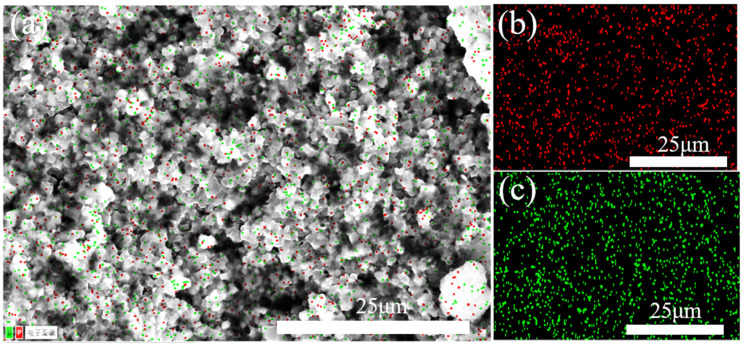
Surface scanning energy spectral distribution of BP prepared at HPHT. (**a**) General EDS map and “电子图像” indicates an “electronic image”; (**b**) phosphorus distribution; (**c**) boron distribution.

In order to determine the elemental distribution of the boron phosphide samples, we used an energy spectrometer (EDS) for the analysis. As shown in Figure 11, the red part is the distribution of phosphorus, and the green part is the distribution of boron; we can see that the elements of phosphorus and boron are uniformly distributed on the crystal, and the distribution of boron will be slightly less than that of phosphorus because the X-ray is weak at detecting elements with small atomic numbers, so the detected boron elements are less compared with the actual ones. The scale of the image is 25 microns, indicating that this is an elemental distribution analysis on a micron scale. Overall, this EDS elemental distribution map shows a uniform distribution of phosphorus and boron elements in the boron phosphide material, indicating that the preparation process of the material is more successful, with uniform elemental distribution and no obvious segregation.

## 6. Conclusions

In this work, we have successfully prepared boron phosphide materials by a one-step method using amorphous boron powder and amorphous red phosphorus at HPHT, which has the advantages of a short preparation time, simple operation steps and high sample purity. In our experiments, we successfully synthesized the boron phosphide material under different pressure and temperature conditions. Scanning electron microscopy results show that a small portion of the grain size is close to the micro–nanostructure, and synthesized boron phosphide is a multi-sized polycrystalline material. EDS analysis shows that the elemental distribution of the sample is relatively uniform. The conductivity of the tested samples showed that the conductivity and Seebeck coefficient of boron phosphide increased significantly with increasing temperature, suggesting that boron phosphide has semiconducting properties, which was consistent with the theoretically calculated conclusion. In the theoretical calculations based on XRD patterns, the partial density of states for each orbital for boron phosphide gives a qualitative explanation for the experimental phenomenon of increasing conductivity with increasing temperature. We also find that high pressure reduces the lattice constants of boron phosphide material, leading to a decrease in its forbidden bandwidth and an increase in conductivity. This suggests pressure can be used as an efficient control method to improve conductivity by modulating the band structure. In the future, we intend to further explore the peak of its electrical properties under higher pressure and conduct mechanical property tests. To reach the peak hardness of the appropriate ratio, we further try to add metal Mg or Al to improve the hardness of BP and explore it.

## Figures and Tables

**Table 1:** Structural information of *F*-43m BP calculated based on XRD pattern at 1 bar and 4 GPa.

Phase	Pressure	Lattice Parameters (Å)	Atom	Atomic Coordinates		
				x	y	z
*F*-43m	1 bar	a = b = c = 4.54659	B (4a)	0.0000	0.0000	0.0000
		α = β = γ = 90	P (4c)	0.2500	0.2500	0.2500
	4 GPa	a = b = c = 4.51092	B (4a)	0.0000	0.0000	0.0000
		α = β = γ = 90	P (4c)	0.2500	0.2500	0.2500

**Table 2:** Calculated lattice constant and band gap are compared to the reported values.

Reference	Lattice Constant a (Å)	Band Gap (eV)
This work (1 bar)	4.54659	1.9810
This work (4 GPa)	4.51092	1.9479
Ref. [25]	4.5400	2.1
Refs. [26,27]	4.538	2.01

## Data Availability

Data are contained within the article.

## References

[1] Wang Z., Yao Y., Zhu L., Liu H., Iitaka T., Wang H., Ma Y. (2014). Metallization and superconductivity of BeH_2_ under high pressure. J. Chem. Phys..

[2] Lu C., Li Q., Ma Y., Chen C. (2017). Extraordinary indentation strain stiffening produces superhard tungsten nitrides. Phys. Rev. Lett..

[3] Howie R.T., Guillaume C.L., Scheler T., Goncharov A.F., Gregoryanz E. (2012). Mixed molecular and atomic phase of dense hydrogen. Phys. Rev. Lett..

[4] Drozdov A.P., Eremets M.I., Troyan I.A., Ksenofontov V., Shylin S.I. (2015). Conventional superconductivity at 203 kelvin at high pressures in the sulfur hydride system. Nature.

[5] Jin X., Chen X.-J., Cui T., Mao H.-K., Zhang H., Zhuang Q., Bao K., Zhou D., Liu B., Zhou Q. (2016). Crossover from metal to insulator in dense lithium-rich compound CLi4. Proc. Natl. Acad. Sci. USA.

[6] Zhuang Q., Jin X., Cui T., Zhang D., Li Y., Li X., Bao K., Liu B. (2018). Effect of electrons scattered by optical phonons on superconductivity in *M*H_3_ (*M* = S, Ti, V, Se). Phys. Rev. B.

[7] Jin X., Meng X., He Z., Ma Y., Liu B., Cui T., Zou G., Mao H.-K. (2010). Superconducting high-pressure phases of disilane. Proc. Natl. Acad. Sci. USA.

[8] Yuan Y.-F., Zhang Z.-T., Wang W.-K., Zhou Y.-H., Chen X.-L., An C., Zhang R.-R., Zhou Y., Gu C.-C., Li L. (2018). Pressure-induced enhancement of optoelectronic properties in PtS_2_. Chin. Phys. B.

[9] Zhang D., Jin X., Zhuang Q., Li Y., Yang S., Song L., Liu B., Cui T. (2019). Crystal structures and decomposing of B–P compounds under pressure. Chin. Phys. B.

[10] Muthaiah R., Garg J. (2020). Strain tuned high thermal conductivity in boron phosphide at nanometer length scales—A first-principles study. Phys. Chem. Chem. Phys..

[11] Deb J., Sarkar U. (2021). Boron-nitride and boron-phosphide doped twin-graphene: Applications in electronics and optoelectronics. Appl. Surf. Sci..

[12] Gao S., Yu H., Yang P., Zhang Y., Ma H., Jia X. (2024). Improved thermoelectric properties of SrTiO_3_-based ceramic/CNTs composite synthesized via high-temperature and high-pressure method. Ceram. Int..

[13] Zhao Y., Mao J., Wu Z., Io W.F., Pang S.-Y., Zhao Y., Hao J. (2024). A clean transfer approach to prepare centimetre-scale black phosphorus crystalline multilayers on silicon substrates for field-effect transistors. Nat. Commun..

[14] Li H., Li J., Liu Y., An K., Liu F., Lu J. (2024). Ideal electrodes for monolayer boron phosphide and their device performance. Appl. Surf. Sci..

[15] Shi H.-X., Yang K.-K., Luo J.-W. (2021). Origin of abnormal thermal conductivity in group III–V boron compound semiconductors. Acta Phys. Sin..

[16] Kumashiro Y., Okada Y. (1985). Schottky barrier diodes using thick, well-characterized boron phosphide wafers. Appl. Phys. Lett..

[17] Yu H., Yang P., Jiang L., Li X., Ji W., Gao S., Chen Y., Zhang Y., Ma H., Jia X. (2024). Improvement of thermoelectric properties of xNb:(1 − x)SrTiO_3_ composite ceramics by high-pressure high-temperature synthesis. Ceram. Int..

[18] Li G., Abbott J.K., Brasfield J.D., Liu P., Dale A., Duscher G., Rack P.D., Feigerle C.S. (2015). Structure characterization and strain relief analysis in CVD growth of boron phosphide on silicon carbide. Appl. Surf. Sci..

[19] Tahan Y., Rapaud O., Pradeilles N., Carles P., Maître A., Dine S., Vrel D., Moutaabbid H., Le Godec Y., Genevois C. (2025). Investigating the thermal decomposition of BP into B_12_P_2_: Experimental insights and kinetic modelling at high temperatures. Acta Mater..

[20] Kresse G., Furthmüller J. (1996). Efficient iterative schemes for ab initio total-energy calculations using a plane-wave basis set. Phys. Rev. B.

[21] Perdew J.P., Burke K., Ernzerhof M. (1996). Generalized Gradient Approximation Made Simple. Phys. Rev. Lett..

[22] Blöchl P.E. (1994). Projector augmented-wave method. Phys. Rev. B.

[23] Heyd J., Scuseria G.E., Ernzerhof M. (2003). Hybrid functionals based on a screened Coulomb potential. J. Chem. Phys..

[24] Togo A., Oba F., Tanaka I. (2008). First-principles calculations of the ferroelastic transition between rutile-type and CaCl_2_-type SiO_2_ at high pressures. Phys. Rev. B.

[25] Gui R., Xue Z., Zhou X., Gu C., Ren X., Cheng H., Ma D., Qin J., Liang Y., Yan X. (2020). Strain stiffening, high load-invariant hardness, and electronic anomalies of boron phosphide under pressure. Phys. Rev. B.

[26] Liang W., Zhang L., Xiang X., Wang J., Zhang L., Wu B., Wang Y., Zeng Y., Guan S., Tang Q. (2021). Growth of millimeter-size single-crystal boron phosphide by eutectic melt at 5.0 GPa and 3000 °C. Solid State Commun..

[27] Liang W., Xiang X., Li Q., Liang H., Peng F. (2024). Stability and melting behavior of boron phosphide under high pressure. Chin. Phys. B.

